# Functional architecture and cell wall composition of peltate scales involved in leaf water absorption

**DOI:** 10.3389/fpls.2026.1756403

**Published:** 2026-02-12

**Authors:** Maria Eduarda dos Santos, Jéssica Ferreira de Lima, Denis Coelho de Oliveira, Ana Silvia Franco Pinheiro Moreira

**Affiliations:** 1 Laboratório de Fisiologia Vegetal, Instituto de Biologia, Universidade Federal de Uberlândia (UFU), Uberlândia, Brazil; 2 Laboratório de Anatomia, Desenvolvimento Vegetal e Interações, Instituto de Biologia, Universidade Federal de Uberlândia (UFU), Uberlândia, Brazil

**Keywords:** cell wall dynamics, foliar water uptake, functional anatomy, immunohistochemistry, plant ecophysiology

## Abstract

**Introduction:**

Foliar water uptake (FWU) is a mechanism that contributes to plant water balance, especially in epiphytic environments. Morphological features such as the presence of peltate scales in bromeliads are adaptive traits for FWU. In this study, we investigated which structural and chemical variations in the walls of these trichomes are related to FWU. To address this, we used *Tillandsia loliacea* Mart. ex Schult. & Schult.f., *Tillandsia pohliana* Mez, and *Tillandsia recurvata* (L.) L. (Bromeliaceae) to (i) describe the histochemical and immunocytochemical features of the peltate scales; (ii) verify whether the density and shape of the scales affect the maximum foliar water uptake capacity and the water absorption rate; and (iii) investigate whether water availability in the leaf tissue (relative water content, RWC) influences FWU in these species.

**Methods:**

Cytological, histological, leaf water balance traits and immunocytochemical analyses were combined with FWU measurements.

**Results:**

We showed that the scales of the three study species exhibit similar structures, including total area and central disc area, but differ in shape.Pectins, hemicelluloses, and proteins were found at the junctions between the periclinal and anticlinal walls of the dome and basal cells, which correspond to the points where the scales attach to the ordinary epidermal cells. Lignin was present in the periclinal wall of the leaf epidermis, as were lipids; however, the latter were found only in the outer periclinal wall and extended along the anticlinal walls of the basal cells. Species with more irregularly shaped central discs and lower relative water content absorbed more water through their leaves.

**Discussion:**

While some models suggest pathways for water entry via the scales, in this work we show histochemical features and water balance traits that may favor FWU, an important foliar trait in bromeliads.

## Introduction

Atmospheric bromeliads, such as *Tillandsia* and *Vriesea* species, are considered xerophytes and are characterized by succulent leaves covered with a dense peltate scale covering (Benzing, 1976). These trichomes are adaptive traits of bromeliads, reflecting their habitat, promoting foliar water uptake (FWU) rather than root water absorption (Tomlinson, 1969; Benzing, 1976, 2000). In *Tillandsia*, the peltate scales cover the entire leaf epidermis (Benzing, 1980) and, like other aerial bromeliads without tank formation, have an irregular distribution along the leaf blade. This distribution may vary between leaf faces, with species having more scales on the adaxial than on the abaxial face (Kleindesings et al., 2018). On these taxa, the peltate scale is formed by stalks and shields (Benzing, 1976, 2000; Pierce, 2007). The stalk is formed by an anticlinally elongated axis with three to five arranged cells, in which the basal cells are known as foot cells and the apical cells are the dome cells. The shield is formed by a central disc composed of four central cells surrounded by concentric rings of monolayers of cells from which the wing cells originate (Benzing, 1976; 2000; Papini et al., 2010; Raux et al., 2020). The shield cells are connected to the dome cell, which in turn is connected to the leaf mesophyll via the basal cells. The basal cells of the stalk have an additional cuticle on the lateral walls that directs water flow toward the mesophyll (Raux et al., 2020). Basal cells have semipermeable membranes that prevent the efflux of water from the mesophyll to the dome and shield cells, favoring unidirectional water flow (Raux et al., 2020).

The cell wall traits, such as thickness, seem to determine shield flexibility and, consequently, the shortening movement due to water loss, causing the wings to rise. This movement may depend not only on the cell wall thickness and number (Benzing et al., 1978), but also on the composition of these cell walls. Foliar water absorption occurs mainly via the apoplast, and this movement depends in part on the composition of the cell wall (Li et al., 2023). One of the main component of plant cell walls are pectins, a non-cellulosic polysaccharides rich in galacturonic acid (GalA) (Caffall and Mohnen, 2009), divided into three main domains depending on the types of monosaccharides associated: homogalacturonan (HGs), ramnogalacturonan I (RG-I), and ramnogalacturonan II (RG-II) (Caffall and Mohnen, 2009; Albersheim et al., 2011). HGs are linear polymers of D-galacturonic acid linked to α-(1→4) with 100–200 GalA residues that are synthesized in a high methyl-esterified form and demethyl-esterified by the action of pectin methylesterases (PMEs), leading to changes in cell wall functional traits (Wolf and Greiner, 2012). RG-I has side chains linked via C-4 of the rhamnosyl chain with predominant arabinosyl and galactosyl residues, while RG-II is a more complex type of pectin and is less abundant in the cell wall (Atmodjo et al., 2013). The different degrees of methyl esterification of these molecules can increase their capacity to form gels and absorb water (Albersheim et al., 2011) or, in some cases, contribute to the mechanical support of the cell wall (Hongo et al., 2012). In these contexts, the pectin cell wall composition and dynamics can directly impact water absorption and scale movement in bromeliads.

Another important group of non-cellulosic polysaccharides is the hemicelluloses, which are composed of monosaccharide units linked by β-(1→4) bonds in the equatorial position (Scheller and Ulvskov, 2010). Pentose (xylose and arabinose) and hexose (mannose, glucose, and galactose) side chains branch from the main backbone (Chandra et al., 2007), giving rise to the major hemicellulose classes in the cell wall: xyloglucans, xylans, and mannans (Chundawat et al., 2011). Xyloglucans are generally the most abundant, contributing to cell wall loosening and expansion (Lima et al., 2025). In addition to hemicelluloses, proteins such as extensins may also be present in the cell wall. Extensins are hydroxyproline-rich glycoproteins involved in various physiological processes, including cell growth and differentiation, wound healing, and defense against pathogens, and they confer rigidity to the cell wall through cross-linking mediated by tyrosine residues (Castilleux et al., 2021). In fact, these non-cellulosic components, together with cell wall-associated proteins, can be decisive for regulating water flux in bromeliad scales. Understanding their distribution and function enables a deeper analysis of the mechanisms of water absorption and of how this functional trait becomes adaptive within the taxa.

Given the efficiency of water uptake by the apoplast of peltate scales, we suppose an intimate relationship with the chemical composition of cell walls. Herein, we propose to understand which structural and chemical characteristics of these specialized trichomes favor leaf water absorption in *Tillandsia* species. We expect to find differences in the chemical composition of the wall along the peltate scale, relating to the degree of methyl esterification of pectins, as well as the presence of hemicelluloses and structural proteins such as extensins, with specific mechanical and hydrophilic properties of the shield and stalk cells. Specifically, the objectives of this study are (a) to determine whether structural characteristics, such as the density and shape of the scales, affect the rate of water absorption and its speed; (b) to describe histochemical and immunocytochemical characteristics of peltate scales that may be related to scales and apoplastic water flow; and (c) to verify whether there is a relationship between the availability of water in the leaf tissue (saturated water content, and relative water content), the structural characteristics of the peltate scales (the total surface area of the scale, disc area, circularity of the total surface of the scale, and circularity of the disc), and the FWU of these species.

## Materials and methods

### Study material

Individuals of *Tillandsia loliacea* Mart. ex Schult. & Schult.f.*, Tillandsia pohliana* Mez, and *Tillandsia recurvata* (L.) L. were collected from different species of phorophytes found on the perimeter of the Umuarama campus (18° 53’ 8.099” S 48° 15’ 33.023” W), at Universidade Federal de Uberlândia (UFU), located in the urban area of the municipality of Uberlândia, Minas Gerais, between May 2023 and March 2024. According to the Köppen classification, the local climate is Cwa (Alvares et al., 2013), characterized by a dry winter (from May to August) and a hot and dry summer (from October to March). For this study, mature and intact leaves were selected from the middle region of the rosettes, prioritizing the central region of the leaves for the analyses.

### Structural analyses

The scale density was obtained from micrographs of two intermediate leaves from the rosette of every five individuals of each species, using a Leica^®^ SAPO magnifying glass (Leica Microsystems, Germany) set to higher magnification, with a digital camera coupled to a Leica^®^ MC 170 HD and LAS EZ Leica^®^ software (version 3.4.0). The micrographs were obtained in the median region of the abaxial and adaxial surfaces of *T. loliacea* and *T. pohliana. Tillandsia recurvata* has terete leaves with a strong reduction in the adaxial surface, with only the abaxial surface being micrographed. The number of scales per area was determined using ImageJ (version 1.51; National Institutes of Health, USA), and the area was adjusted to the micrograph scale.

The same leaves were fixed in FAA _50_ (formalin, acetic acid, 50% ethyl alcohol, 1:1:18 v/v) and stored in 70% ethanol after 48 h (Johansen, 1940). To observe the shield, 30 peltate scales (n = 30) of each species were detached from the epidermis in the median region by scraping with a razor blade. The material was placed on slides, stained with safranin, and mounted with 50% glycerin. Images of six scales of each leaf were obtained on a Leica^®^ DM1000 LED microscope (Leica Microsystems, Germany). Measurements of the total surface area of the scale, disc area, circularity of the total surface of the scale, and circularity of the disc were performed using ImageJ software. Circularity was obtained considering a 4π* area/perimeter², where a value of 1.0 indicates a perfect circle and values closer to 0.0 indicate an elongated shape (indicative of morphological irregularity). The wing area was obtained as the difference between the total surface area of the scale and the area of the disc.

Scanning electron microscopy was used for structural observation and determination of the arrangement of the scales along the leaf blade. Fragments were removed from the middle region of the leaves and fixed in Karnovsky (4% paraformaldehyde, 0.01 M glutaraldehyde, and 0.2 M phosphate buffer, pH 7.2) (5:3:2, v/v) (Karnovsky, 1965, modified by Kraus and Arduin, 1997), gradually dehydrated in an ethanol series, subjected to CO_2_ critical point drying (EMCPD 300, Leica, Germany) and metallized in gold (20 to 30 nm thickness) using an SCD O50 Sputter Coater (BalTec, USA). The samples were analyzed by SEM (VEGA 3, TESCAN, Czech Republic) at 10 kV.

### Histochemistry

Histochemical tests for pectins, lipid substances, and lignins (generally associated with apoplastic water flow and/or cell wall rigidity) were done in cross-sections obtained with the aid of a razor blade in the middle region of fresh leaves from different individuals (n=3). For pectins, the sections were immersed in an aqueous solution of 0.02% ruthenium red (CAS:11103-72-3) for 5 minutes (Johansen, 1940). For lipid detection, the sections remained in an ethyl solution (80%) of 0.5% Sudan III (CAS 85-86-9) for 30 minutes (Sass, 1951). For lignin detection, a 1% phloroglucinol solution was used, followed by an aqueous hydrochloric acid solution (26.5%) for 5 minutes (Johansen, 1940). To confirm the histolocalization of the lignins, DAPI filter was also used, with an excitation wavelength range of 385–400 nm (Chomicki et al., 2014; Joca et al., 2020). The slides were mounted with 50% glycerin and photomicrographed on a Leica^®^ DM1000 LED microscope (Leica Microsystems, Germany) with a Leica^®^ ICC50W coupled digital camera and using the LAS EZ Leica^®^ software (version 3.4.0). Pink color was considered positive for pectic substances (ruthenium red), yellow-orange or red for lipids (Sudan III), and red for lignin (acidified phloroglucinol).

### Immunocytochemistry

Immunocytochemical assays for pectic, protein, and hemicellulose components of the cell wall were done from fragments of the middle portion of the leaves. The samples were dehydrated in an ethanol series, infiltrated in Historesin^®^ (Leica, Germany) according to the manufacturer’s recommendations, and sectioned (4 µm) in a rotary microtome (YD315, ANCAP, Brazil). For each primary antibody, 4 sections from 2 individuals were analyzed (n= 4 sections per individual) and the experiment was independently repeated twice with different individuals. The sections were immersed in a blocking solution (3% Molico^®^ milk in phosphate-buffered saline - PBS) for 30 minutes and incubated with the following primary monoclonal antibodies (MAbs) diluted 1:5 in PBS for 1 hour: Anti-Pectic polysaccharide (beta-1, 4-galactan) [LM5] Antibody, Anti-Pectic polysaccharide (homogalacturonan) [LM19] Antibody, Anti-Pectic polysaccharide [LM20] Antibody, Anti-Arabinogalactan-protein (AGP) [LM2] Antibody, Anti-Extensin glycoprotein [LM1] Antibody, Anti- Heteromannan [LM21] Antibody, LM6 [(1 →5) α-L-arabinan]), and LM11 (1→4)- β-D- xylan/arabinoxylan, all provided by the Center for Plant Sciences, University of Leeds, United Kingdom. Fragments without primary antibodies were used as controls; in these sections, the secondary antibody was applied under the same conditions as in the experimental samples All sections were washed in PBS and then incubated with a secondary anti-mouse IgG (whole-molecule) antibody conjugated to FITC (Sigma-Aldrich^®^, USA) (1:100 in 3% milk/PBS) in the dark for 2 hours. Before being immersed in blocking solution and incubated with the antibodies, the sections were pretreated with 2 mM CaCl buffer containing 10 µg ml^-1^ pectate lyase (CAS 9032-75-1; Sigma-Aldrich) and 50 mM 3-(cyclohexylamino)-1-propanesulfonic acid (CAPS) (Sigma-Aldrich) for 2 hours. The sections were subsequently washed in PBS, mounted in 50% glycerin, and evaluated using a Leica^®^ DM4000 B LED microscope (Leica Microsystems, Germany) with a digital camera coupled to a Leica^®^ DFC3000G and the software Leica^®^ Application Suite X (version: 1.1.0.12420). FICT and DAPI filters were used, with wavelengths of 450-490 µm, a barrier at 515 nm, and an overlap function to eliminate autofluorescence. DAPI filter was also used as a second control in the function overlap of the microscopy software.

### Fluorescent apoplastic marker

The fluorescent marker Lucifer yellow (LY; CH dilithium salt; Sigma-Aldrich^®^) was used to distinguish the apoplastic pathway of water absorption in the leaves of the three species. Three fresh leaves were collected from three individuals and sealed at the base with paraffin. A total of 100 μl of 1% LY aqueous solution was applied to the abaxial and adaxial surfaces of the middle region of the leaves. For the control, distilled water was applied to both sides of the middle region of the third leaf surface. The samples were kept in a dark and humid chamber (Eller et al., 2016). Cross sections were made with the aid of a razor blade in the region where the marker was applied. The histological slides were mounted in 50% glycerin, and the sections were observed under a fluorescence microscope (DM4000 LED, Leica, Germany) with green excitation (450-490 nm) and a 515 nm barrier filter.

### Water balance

Foliar water uptake (FWU) and leaf water balance variables, saturated water content (SWC), and relative water content (RWC) were measured for five individuals of each species (n= 5). The experiments were performed in the morning, starting at 5:30 am, a time when the plants are naturally exposed to greater atmospheric water availability. One leaf from the middle portion of the rosette (from the third node) of each individual was selected for the experiments. The excised leaves were immediately sealed with Vaseline paste, taken to the laboratory, and weighed (fresh mass, mg) on an analytical balance (Shimadzu AUY 220, Japan). The leaves were subsequently immersed in distilled water and weighed every 15 minutes for 2 h, then every 30 minutes for another 2 h, and finally, after 1 h, until there was no further change in leaf weight. The leaves were dried with an absorbent towel to remove surface water before each weighing (Liang et al., 2009). At the end of the process, the leaves were photographed, and the areas were measured using ImageJ.

Water absorption by the leaves was calculated using differential equations adapted for nonlinear regression, which allow measurement of dynamic foliar water uptake and its variation over time (until the leaves reach saturation). The dynamic water content (C) was calculated using the equation C = (CW-DW)/DW, where (CW) represents the weight of water contained in the leaf and DW represents the weight of the leaf dry matter. The variation in dynamic water content or increase in foliar water absorption was obtained by the equation ΔC = (C-Ci), where C represents the dynamic leaf water content, and Ci represents the initial water content. Thus, the maximum water absorption capacity by the leaf was represented by ΔC_max_, which was defined as the maximum difference between the water saturation content (Cs) and the initial water content in the leaf (ΔC_max_ = Cs-Ci). A differential equation with time-dependent data (t), ΔC = ΔC_max_ (1 - e ^-kt^), was used to estimate the water absorption rate (k) (Liang et al., 2009). The equations were obtained using SigmaPlot software (version 15.1; Systat Software, USA).

The SWC of the leaves was obtained by dividing the difference between the turgid mass (TW) and the dry mass (DW) by the dry mass, where SWC = (TW-DW)/DW (Ogburn and Edwards, 2012). The RWC was obtained as the difference between the fresh mass (FW) and the dry mass (DW) divided by the difference between the turgid mass (TW) and the fresh mass (FW), multiplied by 100%, where RWC = (FW - DW)/(TW- FW) *100% (Turner, 1981).

### Data analysis

One-way analysis of variance (ANOVA) was performed to determine differences between species in morphological, foliar water uptake, and water balance variables, which met the assumptions of normality and homogeneity of variance as assessed by the Shapiro-Wilk and Levene tests, respectively. The nonparametric data (total area of the peltate scale, total circularity of the peltate scale, circularity of the peltate scale disc, wing area of the peltate scale, saturated water content, and maximum foliar water absorption) were analyzed using the Kruskal-Wallis test. The analyses were followed by the Tukey HSD test for multiple comparisons of means. All analyses were conducted using the R programming language (R Core Team, version 4.4.3; Austria).

The relationships between foliar water absorption, morphological attributes (the total surface area of the scale, disc area, circularity of the total surface of the scale, and circularity of the disc), and aspects of the species’ water balance (saturated water content, and relative water content) were investigated using simple linear regression models in GraphPad Prism, Inc. (version 8.0.1, USA). The models were constructed based on the mean number of scales per leaf, corresponding to the number of sample units measured for aspects of foliar water uptake and water balance.

## Results

### Structure of peltate scales

**Figure 1:**
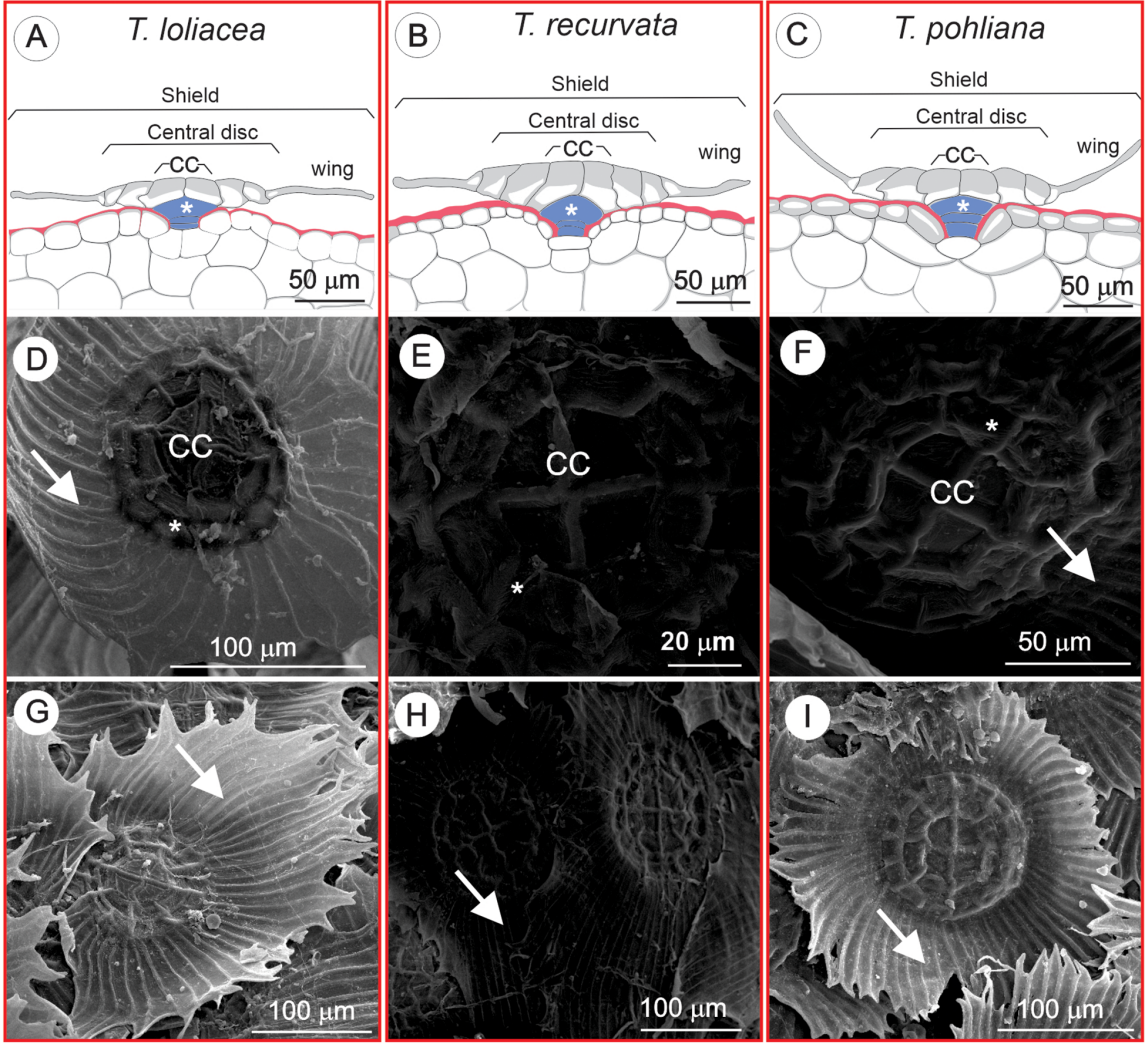
Peltate scales of *Tillandsia loliacea*, *T. recurvata*, and *T. pohliana* collected in the municipality of Uberlândia, Minas Gerais, Brazil. **(A-C)** Schematic of cross-section showing the shield composed of the central disc and the wing. The central disc is formed by central cells (CC) surrounded by the ring cells. The shield is connected to the epidermis by the stalk (blue), composed of the dome cells (*) and two basal cells. **(D-F)** Scanning electron microscopy of the central disc with four central cells (CC) surrounded by concentric rings (*) from which the wings originate (white arrows). **(G-I)** Scanning electron microscopy of the wings (white arrows) of *T. loliacea* and *T. pohliana* with a greater inclination in relation to the leaf surface, while **(F)** the wings in *T. recurvata* are more parallel to the leaf surface.

The scales of the three species have a similar structure (**Figure 1**). The stalks are supported by one to two basal cells, followed by the dome (**Figures 1A-C**). The shield is formed by the central disc, which is composed of four central cells surrounded by concentric rings of monolayer cells from which the wings originate (**Figures 1D-F**). The scale wings of *T. loliacea* and *T. pohliana* were more inclined than the leaf surface, whereas the scales of *T. recurvata* were parallel to the surface (**Figures 1G-I**). The three species had similar total scale areas and disc areas (**Table 1**). *Tillandsia loliacea* had lower total-scale circularity than *T. recurvata* and *T. pohliana*, while disc circularity was similar among the species; *T. pohliana* had lower disc circularity compared with *T. recurvata. Tillandsia loliacea* had a larger wing area than *T. recurvata* plants. The density of peltate scales on the abaxial surface of the leaves was lower in *T. pohliana* and greater in *T. recurvata. Tillandsia loliacea* did not vary in scale density on the leaf adaxial surface, but compared with *T. pohliana*, its plants had lower peltate-scale density on the adaxial surface.

**Table 1:** Structural attributes of the peltate scales of *Tillandsia loliacea*, *T. recurvata*, and *Tillandsia pohliana* (n=30, mean ± standard deviation).

Species	Total area of shield (mm²)	Total circularity of the shield	Area of the central disc (mm²)	Circularity of the central disc	Area of the wing (mm²)	Abaxial density of peltate scales (scales mm^-^²)	Adaxial density of peltate scales (scales mm-²)
*T. recurvata*	0.08 ± 0.01	0.58 ± 0.14a	0.01 ± 0.002	0.997 ± 0.003a	0.06 ± 0.01b	35.56 ± 7, 68a.	**--**
*T. loliacea*	0.09 ± 0.01	0.26 ± 0.08b	0.01 ± 0.002	0.995 ± 0.009ab	0.08 ± 0.01a	33.46 ± 6.40ab	32.70 ± 6.06b
*T. pohliana*	0.09 ± 0.02	0.62 ± 0.17a	0.01 ± 0.002	0.992 ± 0.007b	0.07 ± 0.02ab	28.53 ± 6.91b	40.97 ± 10.62a
F or χ² values	χ² = 4.79	χ² = 52.91	F = 3.17	χ² = 9.70	χ² = 7.46	F = 5.09	F = 9.52
p value	> 0.05	< 0.0001	> 0.05	< 0.01	< 0.05	< 0.01	< 0.01

Data were analyzed using one-way ANOVA (F statistic) when assumptions were met; otherwise, the Kruskal- Wallis test (χ²) was applied, in both cases, a multiple comparison *post hoc* test was applied. F or χ² values and the corresponding p-values are presented.

Different letters indicate differences among species according to Tukey’s test at the 5% significance level.

### Chemical composition of the cell wall

**Figure 2:**
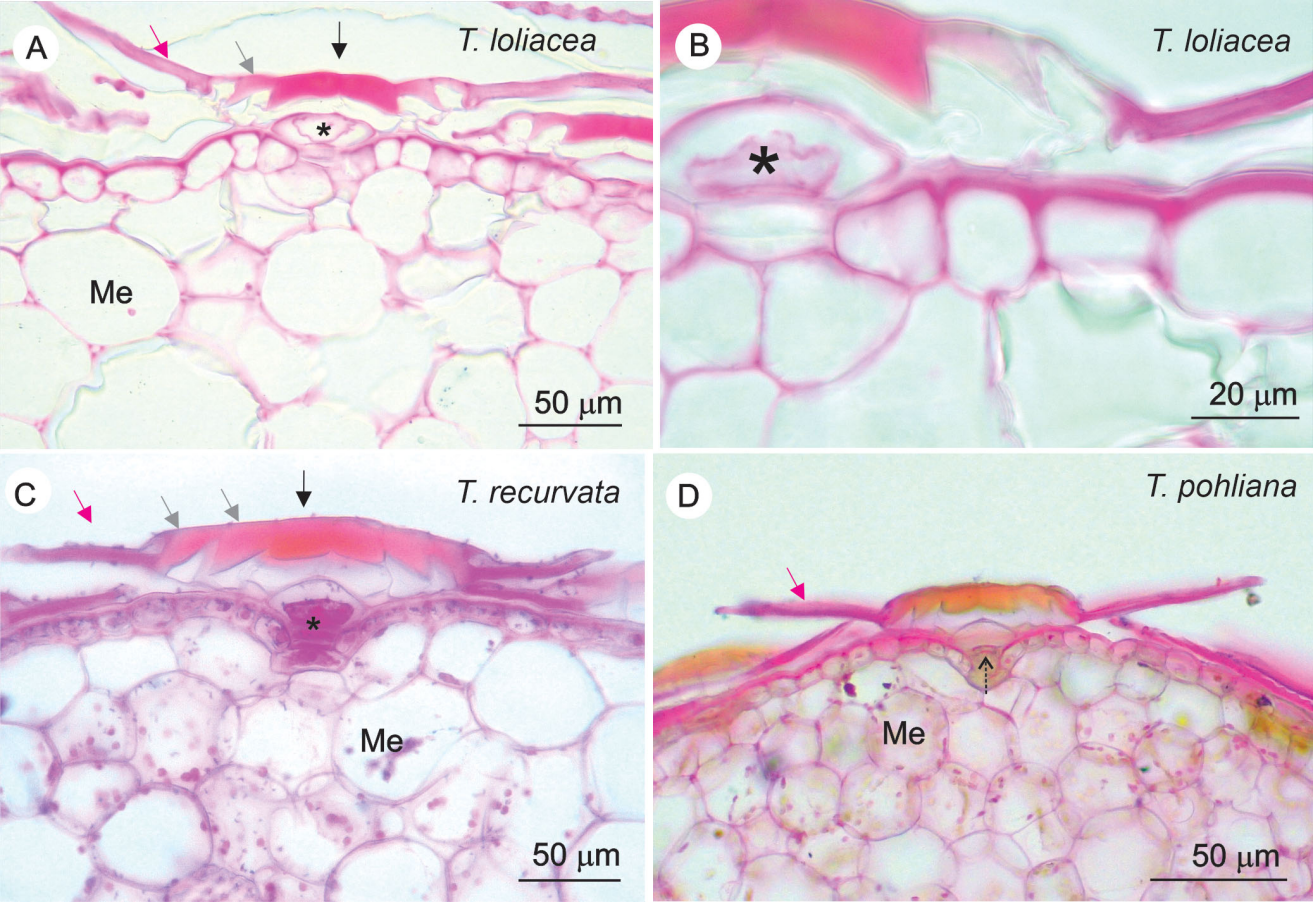
Histolocalization of pectins in the peltate scales of *Tillandsia loliacea*, *T. recurvata*, and *T. pohliana* leaves. The pectins were detected using ruthenium red, with a positive reaction given by the pink color. **(A, B)** *T. loliacea*; pectins were present in the stalk, including the dome cells (*) and in the shield, including central cells (black arrow), ring cells (gray arrow), and wings (pink arrow). **(C)** *T. recurvata*; intense staining in the cellular content of the stalk (*), in addition to the markings in the shield walls, which, like *T. loliacea*, included the central cells (black arrow), ring cells (gray arrow), and wings (pink arrow). **(D)** *T. pohliana*; slight staining of the periclinal walls of the stalk cells (dotted arrow) and intense staining of the wings (pink arrow). Me, mesophyll.

The peltate scales of *T. loliacea*, *T. pohliana*, and *T. recurvata* showed a primary wall composed of pectins, hemicelluloses, and proteins and impregnated with lipids and lignin. The histochemical detection of pectins was similar in the scales of the three species (**Table 2**; **Figure 2**). In *T. loliacea* stalks, pectins were present in the dome cell walls (**Figures 2A, B**). In *T. recurvata*, intense staining was detected inside the stalk cells (**Figure 2C**). In *T. pohliana*, slight staining of the periclinal walls of the stalk cells was detected (**Figure 2D**). In the shields, pectins were detected on the walls of the central cell (except for *T. pohlinana)*, which showed mild staining (**Figure 2D**), in the cells of the rings around the central cells (including in the junctions with the epidermal cells), and with intense reaction on the wings.

**Table 2:** Distribution of different types of cell wall pectins in the epidermis and peltate scales of *Tillandsia loliacea*, *T. recurvata*, and *T. pohliana* leaves.

Species/location	Ruthenium red	LM19	LM20	LM5	LM6
T. loliacea					
Epidermis	+	+	+	+	+
Central disc cells	+	+	+	+	-
Wings	+	+	+	-	-
Dome cells	+	+	+	+	+
Basal cells	+	+	+	-	-
T. recurvata					
Epidermis	+	+	+	+	+
Central disc cells	+	+	+	+	-
Wings	+	+	+	-	-
Dome cells	+	+	+	+	+
Basal cells	+	+	+	-	-
T. pohliana					
Epidermis	+	+	+	+	+
Central disc cells	+	+	+	-	-
Wings	+	+	+	-	-
Dome cells	+	+	+	+	+
Basal cells	+	+	+	-	-

The positive (+) and negative (-) symbols indicate the presence or absence of each type of pectin. Pectins were detected by histochemistry (ruthenium red) and wall immunocytochemistry. They were labeled low methyl-esterified homogalacturonans (LM19 antibody), high methyl-esterified homogalacturonans (LM20), galactans (LM5), and arabinans (LM6).

**Figure 3:**
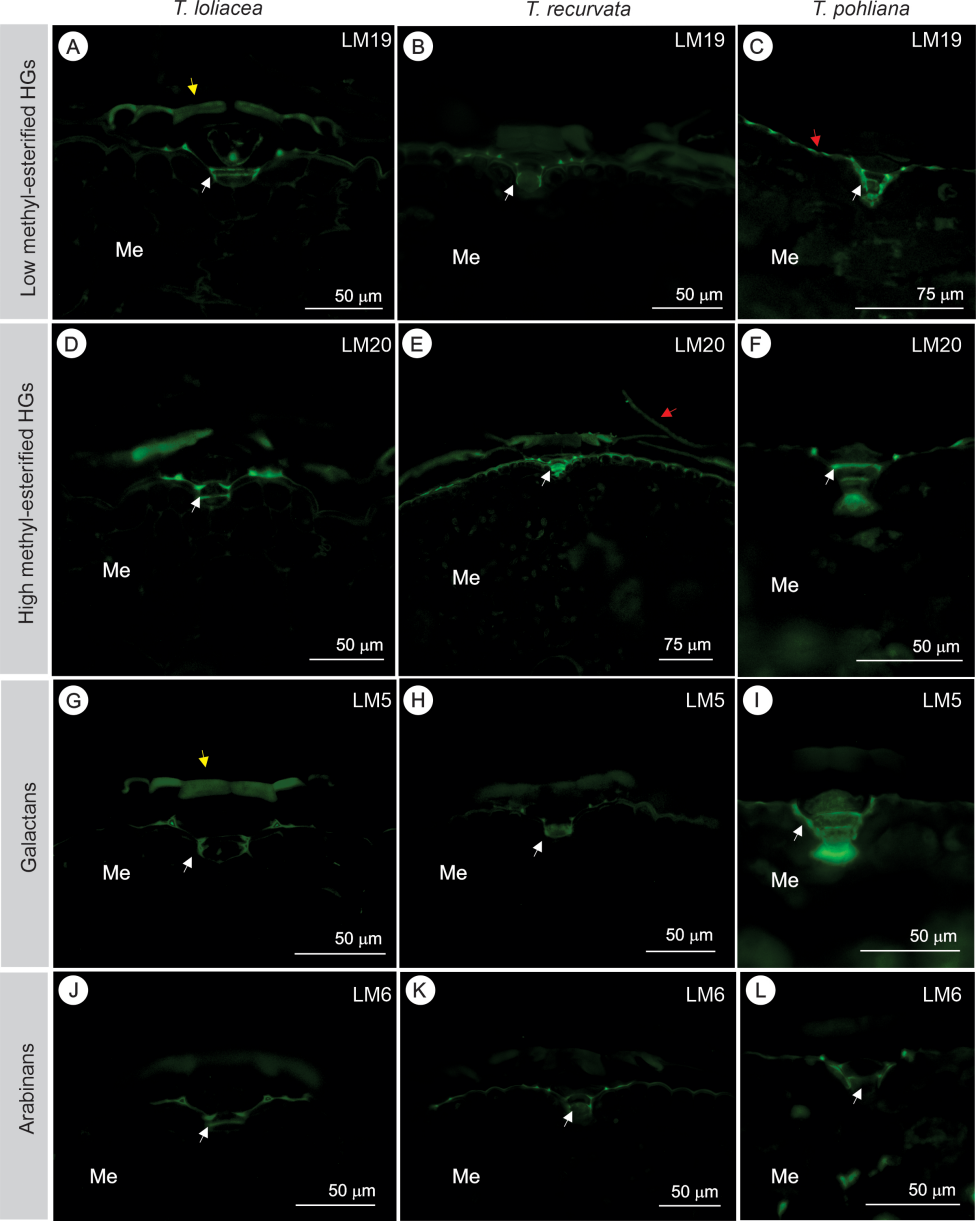
Different types of pectins in the peltate scales of *Tillandsia loliacea* (first column), *T. recurvata* (second column), and *Tillandsia pohliana* (third column) leaves. **(A-C)** Homogalacturonans (HGs) with low methyl-esterification (anti-pectic polysaccharide (homogalacturonan) [LM19] antibody), and **(D-F)** high methyl-esterification (anti-pectic polysaccharide [LM20] antibody). Intense staining in the stalk cells (white arrow), areas of contact with the epidermis, and the insertion points of the wings. The yellow arrows indicate intense staining in the central cells of the disc, and the red arrows indicate intense staining in the wings. **(G–I)** Galatans (anti-pectic polysaccharide (beta-1, 4-galactan) [LM5] antibody) in the stalk cell walls (white arrows) and junctions with the epidermis. In *T. loliacea* **(G)**, galactans were also present on the external surface of the central (yellow arrow) and ring cells of the central disc. The white arrows indicate intense staining in the stalk cells. **(J-L)** Arabinans were labeled with LM6 [(1 → 5) α-L-arabinan]) in stalk cells (white arrows) and in the areas of contact with the epidermis. The white arrows indicate intense staining in the stalk cells. Me, mesophyll.

In addition to histochemical tests, immunocytochemical assays showed different types of pectins in the peltate scales (**Figure 3**). Epitopes of HGs with low methyl-esterified groups were detected in the stalks (basal and dome cells) of the three species, as well as in the epidermis in contact with the peltate scale (**Figures 3A, C**). In *T. loliacea*, epitopes of low methyl-esterified HGs were also detected in the external periclinal walls of the disc (**Figure 3A**), and in *T. pohliana* they were also detected in the wings (**Figure 3C**). Similar results were found for epitopes of high methyl-esterified HGs (**Figures 3D-F**). However, in *T. recurvata*, highly methyl-esterified HGs filled the stalk cells and were more evident in the wings (**Figure 3E**). Epitopes of galactans were detected mainly in the cell wall of the basal cells of the stalk and in the cell junctions with the epidermis (**Figures 3G, I**). In *T. loliacea* and *T. recurvata*, galactans were also present in the upper part of the central and ring cells, where the wings were inserted (**Figures 3G, H**). Epitopes of arabinans were evident mainly in the cells of the stalk and in areas of contact with the epidermis (**Figures 3J-L**).

**Figure 4:**
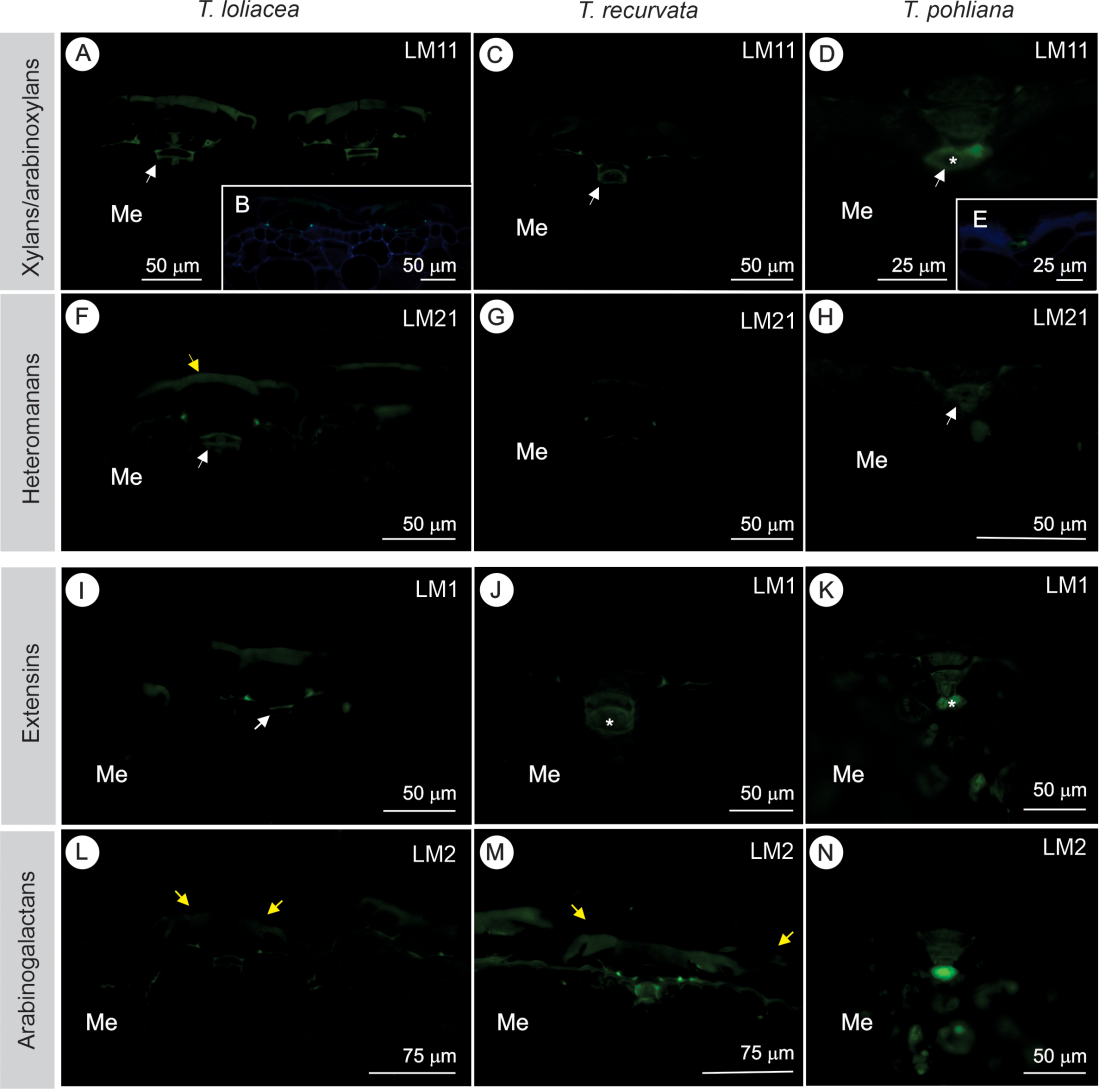
Wall immunocytochemistry for hemicelluloses (xylans/arabinoxylans and heteromannans) and proteins (extensins and arabinogalactans - AGPs) in the peltate scales of *Tillandsia loliacea* (first column), *T. recurvata* (second column), and *Tillandsia pohliana* (third column) leaves. **(A-E)** Xylans and arabinoxylans (LM11 (1→4) - β-D-xylan/arabinoxylan) in the stalk cells (white arrows) and at the insertion points of the scale with the epidermis. In *T. pohliana*, xylans and arabinoxylans were also marked as basal cell content (*). **(B, C)** show immunofluorescence images overlaid with a DAPI filter (blue color), reducing autofluorescence and confirming positive results.**(F-H)** Heteromannans (Antibody, Anti- Heteromannan [LM21] Antibody) are also present in the stalk cells (white arrows) and in the insertion points of the scale with the epidermis. In *T. loliacea*, details of the light staining for hemicelluloses in the outer periclinal walls of the central (yellow arrow) and ring cells of the central disc (AB, F). **(I-K)** Extensins (anti-extensin glycoprotein [LM1]) were labeled in the stalk cells (white arrows) and in the junctions with the epidermis. Details of the extensins as the cellular content of stalk cells (*) in *T. recurvata* and *T. pohliana*. **(L-N)** Arabinogalactans (Antibody, Anti-Arabinogalactan-protein (AGP) [LM2]) were labeled in the ring cells of the central disc (yellow arrows) in *T. loliacea* and *T. pohliana*. Me, mesophyll.

**Figure 5:**
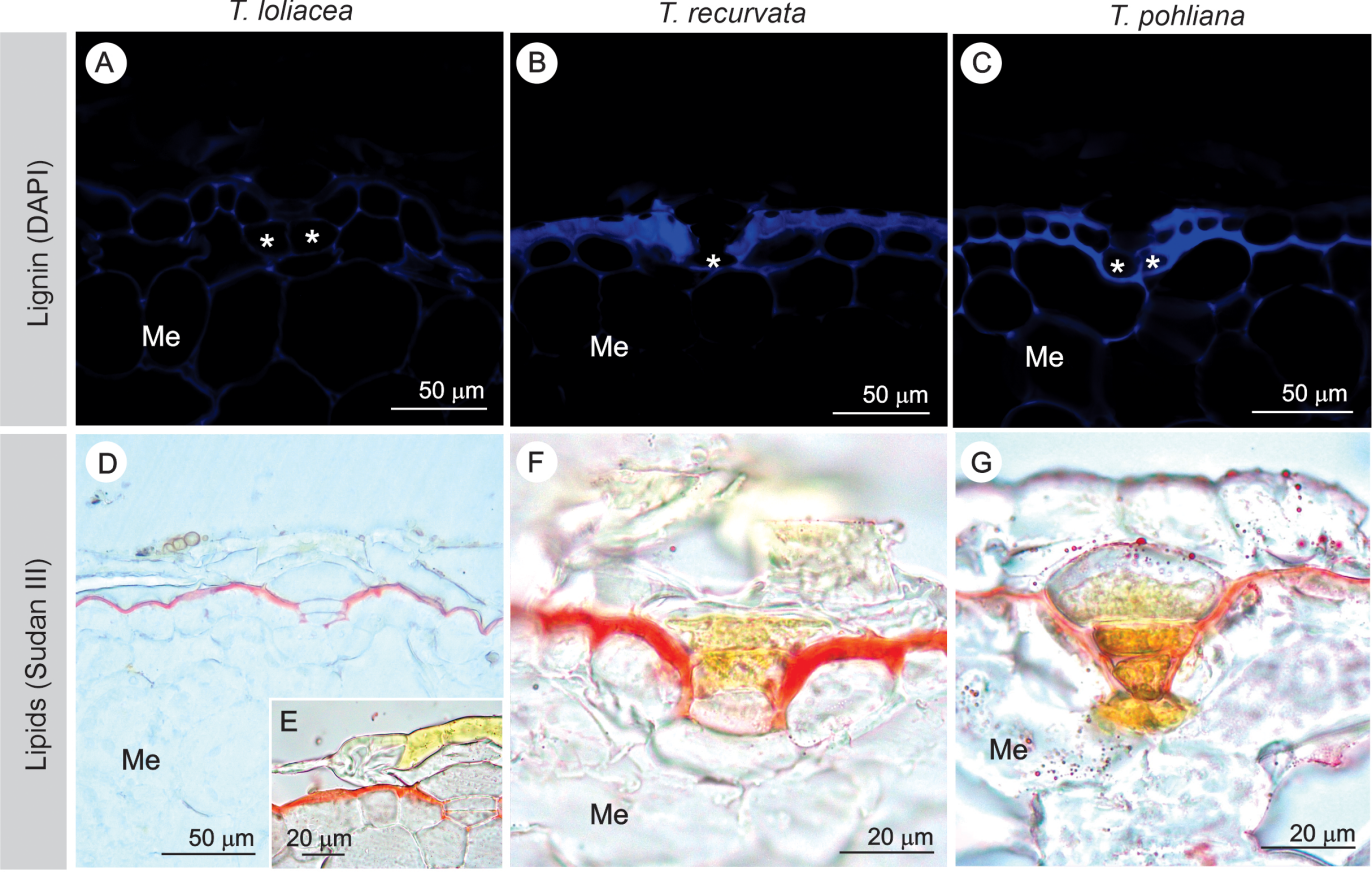
Lignin and lipid impregnation in the cell walls of the peltate scales of *Tillandsia loliacea*, *T. recurvata*, and *T. pohliana* leaves. **(A-C)** The presence of lignin was detected in the epidermal walls and basal cells (*) of the stalks of the three species. **(D-G)** Lipid impregnation was observed in the cuticle, extending through the anticlinal walls of the stalk. Details of the thickened cuticle in *T. recurvata*. Me, mesophyll.

Epitopes of arabinoxylans, heteromannans, extensins, and AGPs, as well as lipids and lignin, were also detected in the cell walls of the ordinary cells of the epidermis and in the peltate scales of the three studied species (**Table 3**, **Figures 4**, **5**). Epitopes of hemicelluloses (xylans/arabinoxylans and heteromannans), AGPs and extensins were detected in similar locations (**Table 3**; **Figure 4**). Epitopes of arabinoxylans (**Figures 4A-E**) and heteromannans (**Figures 4F-H**) were detected in the cell junctions of the stalk cells and at the insertion points of the scale with the ordinary cells of the epidermis. In *T. loliacea*, slight staining was observed in the outer periclinal walls of the central and ring cells of the central disc (**Figures 4A, B, F**). In *T. pohiliana*, epitopes of arabinoxylans were detected in the stalk cells (**Figures 4D, E**). Among the cell wall proteins evaluated in this study, extensins (**Figures 4I-K**) were detected in stalk cells and at cell junctions with ordinary epidermal cells. In T*. loliaceae*, they were labelled only in the outer periclinal walls of the disc (only in *T. loliacea*, **Figure 4I**). In *T. recurvata* and *T. pohliana*, extensins were also labeled in the stalk cells (**Figures 4J, K**). Arabinogalactans (AGPs, **Figures 4L-N**) were labeled in stalk cells, in the central disc, and in the cell junctions with the epidermis. In the central disc, despite the slight staining in the central cells, they were predominant in the ring cells, both in *T. loliacea* (**Figure 4L**) and in *T. recurvata* (**Figure 4M**). *T. pohliana* presented AGPs only in the stalk cells and as the cellular content of the basal cells (**Figure 4N**).

**Table 3:** Presence of hemicelluloses, proteins, lipids, and lignin in the cell walls of the epidermis and peltate scales of *Tillandsia loliacea*, *T. recurvata*, and *T. pohliana* leaves.

Species/location	LM11	LM21	LM1	LM2	Lignin	Lipids
T. loliacea						
Epidermis	+	+	+	+	-	-
Central disc cells	+	+	+	+	-	-
Wings	-	-	-	-	-	-
Dome cells	+	+	+	+	-	+
Basal cells	+	+	-	-	+	+
T. recurvata						
Epidermis	+	+	+	+	-	-
Central disc cells	-	-	-	+	-	-
Wings	-	-	-	-	-	-
Dome cells	+	+	+	+	-	+
Basal cells	+	+	+	-	+	-
T. pohliana						
Epidermis	+	+	+	+	-	-
Central disc cells	-	-	-	-	-	-
Wings	-	-	-	-	-	-
Dome cells	+	+	+	+	-	+
Basal cells	+	+	+	+	+	–

They were stained by wall immunocytochemistry; xylans and arabinoxylans (hemicelluloses, LM11 antibody), heteromannans (hemicellulose, LM21 antibody), extensins (wall proteins, LM1 antibody), and arabinogalactans (wall proteins, LM2 antibody). Histochemistry revealed lignin (DAPI and acid phloroglucinol) and lipid (Sudan III) impregnation. The positive (+) and negative (-) symbols indicate the presence and absence in the different tissues.

Lignin impregnation was detected along the epidermal walls in the three species (**Figures 5A-C**), with thickened walls in *T. recurvata* and *T. pohliana* (**Figures 5B, C**). Lignin was also observed in the stalk cells of the three species, mainly in basal cells. Lipids were detected in the cuticle (**Figures 5D-G**), extending along the sides (anticlinal walls) of the stalk cells. In *T. loliacea*, lipids were also present at the junctions of the dome cells with the basal cells (**Figure 5E**). In *T. recurvata* (**Figure 5F**), the cuticle appeared thicker than that in *T. loliacea* (**Figures 5D, E**) and *T. pohliana* (**Figure 5G**).

### Water balance of leaves and water absorption capacity

**Figure 6:**
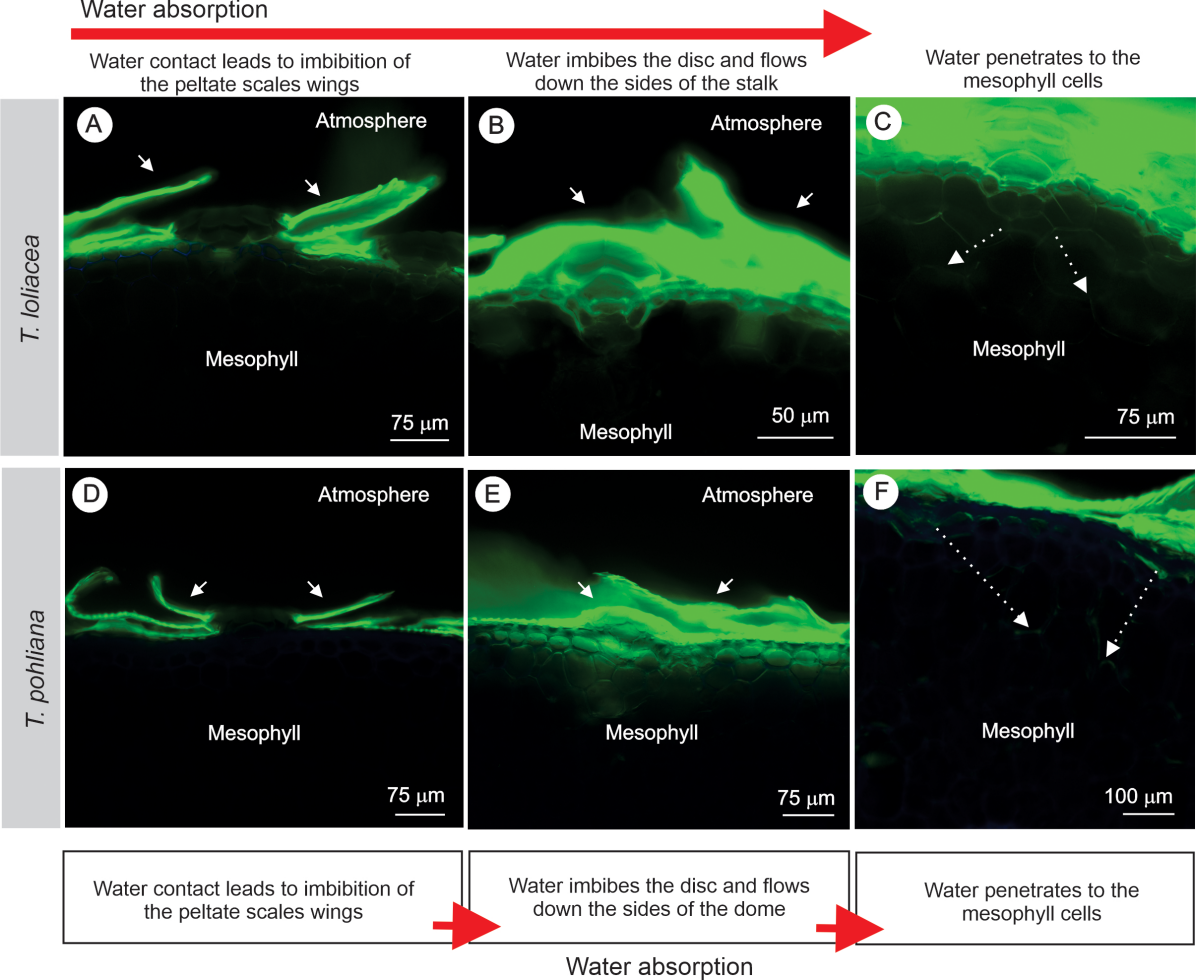
Fluorescence microscopy using the fluorescent apoplastic marker Lucifer Yellow to confirm water absorption by the peltate scales of *Tillandsia loliacea*, *T. recurvata*, and *Tillandsia pohliana* leaves. **(A-C)** *Tillandsia loliacea*. **(D-F)** *Tillandsia pohliana.* The images were organized so that water absorption over time is shown in each row. **(A, D)** In the first column, the initial contact with the marker highlights the high-water affinity of the wings (white arrows). **(B, E)** In the second stage, the disc cells become embedded (white arrows). **(C, F)** In the final column, water diffuses through the mesophyll (white arrows).

An experiment with LY apoplastic marker (**Figure 6**) revealed the high affinity of the wings for water (**Figure 6**). The wings are first embedded by water (**Figures 6A, D**). When the leaves are in contact with the LY solution for a longer period, it is possible to observe that the entire scale is soaked with water (**Figures 6B, E**). This water enters through the dome cells near the epidermal cells. Afterward, the water reaches the mesophyll (**Figures 6C, F**). Among the three species analyzed, individuals of *T. loliacea* and *T. recurvata* had lower RWC values and higher SWC values (**Table 4**). The highest maximum foliar water uptake capacity was obtained for *T. loliacea* plants. *Tillandsia pohliana* plants had higher RWC values and lower SWC values, and absorbed little water by the leaves (C_max_). The velocity of foliar water uptake (K) was the same for the three species investigated (**Table 4**).

**Table 4:** Water balance of Tillandsia loliacea, T. recurvata, and Tillandsia pohliana leaves.

Species	RWC (%)	SWC	C_max_ (g cm-² AF)	k
*T. recurvata*	27.39 ± 7.10b	12.37 ± 3.39a	38.70 ± 15.92 a	0.01 ± 0.004
*T. loliacea*	36.25 ± 3.92b	11.40 ± 0.36a	45.01 ± 11.02a	0.006 ± 0.004
*T. pohliana*	50.46 ± 4.34a	6.61 ± 0.29b	3.31 ± 0.71b	0.01 ± 0.02
F and χ² values	F = 23.97	χ^2^ = 9.5	χ^2^ = 9.98	F = 2.11
p values	< 0.0001	< 0.01	< 0.01	> 0.05

Data were analyzed using one-way ANOVA (F statistic) when assumptions were met; otherwise, the Kruskal- Wallis test (χ²) was applied, in both cases, a multiple comparison *post hoc* test was applied. F or χ² values and the corresponding p-values are presented.

The relative water content (RWC), saturation water content (SWC), foliar water uptake capacity (C_max_), and the velocity of foliar water uptake (k) were evaluated during the morning. Different letters indicate differences among species according to Tukey’s test at the 5% significance level.

**Figure 7:**
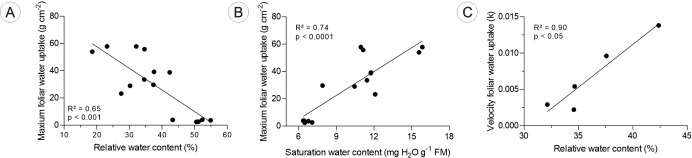
Relationships between maximum foliar water uptake capacity (C_max_) and the velocity of foliar water uptake k. with the relative water content (RWC) and the saturation water content (SWC) of the *Tillandsia loliacea*, *T. recurvata*, and *T. pohliana* leaves. In **(A, B)**, all the individuals from the three species were included. In **(C)**, only individuals of *T. loliacea* were considered. The x-axes indicate leaf water status (RWC or SWC), the y-axes show water uptake traits, and the trends reflect the anatomical and physiological functional traits of the species, illustrating how leaf structure influences water uptake under varying hydration conditions.

Considering all the *Tillandsia* plants studied here, the maximum foliar water uptake capacity (C_max_) decreased with increasing relative water content (RWC) (**Figure 7A**), whereas the saturation water content (SWC) positively influenced the C_max_ (**Figure 7B**). A positive relationship with the velocity of foliar water uptake (k) was found only for *T. loliacea* plants (**Figure 7C**), where k was directly proportional to the increase in the RWC of the leaves. The other relationships were not significant, and the results are available in the **Supplementary Material** (**Supplementary Material 1**).

**Figure 8:**
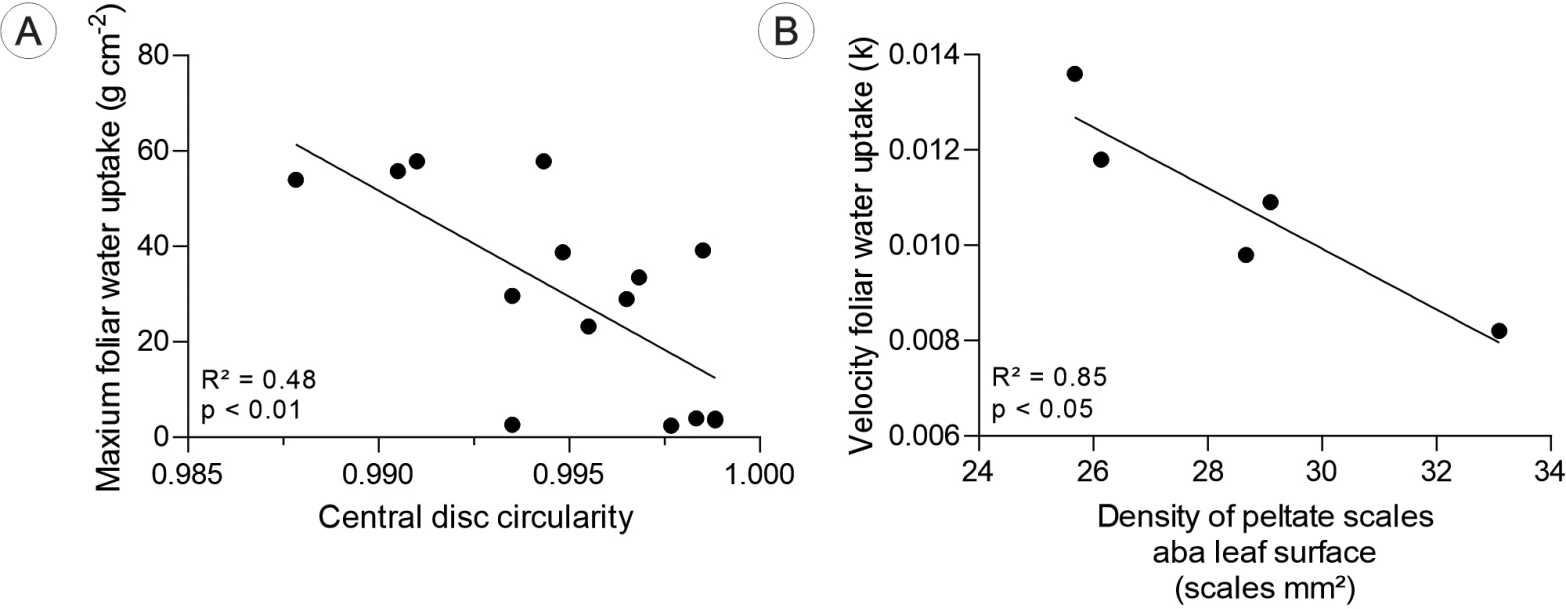
Relationships were established between the maximum foliar water uptake capacity (C_max_) and some structural traits of the peltate scales of *Tillandsia loliacea*, *T. recurvata*, and *T. pohliana* leaves. **(A)** The relationship between C_max_ and the circularity of the central disc was significant when all the plants of the three species were included. **(B)** k was negatively related to the density of the scales on the abaxial surface of the leaves when only *T. pohliana* plants were analyzed. The x-axes indicate structural traits, the y- axes show water uptake traits, and the trends reflect anatomical and physiological characteristics of the species, illustrating how peltate scale structure influences water absorption.

From a structural point of view, the FWU was influenced by the shape of the peltate scales (**Figures 8A, B**). Individuals with lower disc circularity values, i.e., more elongated or misshapen discs, had greater maximum foliar water uptake capacity, whereas those with discs closer to perfect circularity (1.0) had lower C_max_ values (**Figure 8A**). Individuals of *T. pohliana* with a lower density of peltate scales on the abaxial surface of the leaves showed higher velocity of foliar water uptake (**Figure 8B**). The other relationships were not significant, and the results are available in the **Supplementary Material** (**Supplementary Material 1**).

## Discussion

The water flow through the peltate scales of the three *Tillandsia* species studied results from interactions among their architecture, cell wall composition, and physicochemical properties that direct water flow across the leaf surface. The coexistence of rigid and flexible zones, modulated by pectins, hemicelluloses, wall proteins, lipids, and lignin, creates a dynamic functional system capable of capturing, conducting, and storing water in a manner adjusted to atmospheric conditions.

### Distribution and structure of peltate scales

The development of peltate scales in bromeliads involves complex cellular processes that support their physiological efficiency. This trichome, typical of bromeliads, originates from successive periclinal divisions of the protoderm, forming specialized cells that constitute the stalk (dome and basal cells) and the shield (central disc and wings). Its development process ends with programmed cell death of the shield cells when the scale reaches maturity (Papini et al., 2010; Ballego-Campos et al., 2020). On the peltate scales, each cell appears to have a distinct wall composition, with a cellulosic network (John and Hasenstein, 2017) surrounded by a non-cellulosic matrix of pectins, hemicellulose, and proteins, with associated lipids and lignin deposition. The chemical profile of cell walls, associated with structural variation and the density of peltate scales, is an important adaptive strategy in bromeliads, favoring water absorption (Benzing, 1976; Varadarajan and Gilmartin, 1987; Papini et al., 2010).

The density and structure of peltate scales in bromeliad leaves are closely related to their absorption capacity, protection, and thermal regulation functions. The high density of peltate scales observed in xeromorphic and/or “atmospheric” species not only enhances the absorption of water and nutrients but also helps reduce the entry of toxic elements (Palhares et al., 2025) and water loss by transpiration but also reflects excess solar radiation, preventing photoinhibition (Benzing, 1976). With regard to water absorption, the overlapping of scales and the presence of thick marginal wings increase leaf cover and water retention capacity (Varadarajan and Gilmartin, 1987). The scales can secrete polysaccharides that favor osmotic processes and facilitate water absorption under low-humidity conditions (Benzing et al., 1976; Papini et al., 2010), further reinforcing their multifunctional role.

The structural and density variations in the peltate scales recorded among the three *Tillandsia* species studied here indicate distinct strategies for maintaining the leaf water balance. *Tillandsia recurvata* showed a higher density of scales on the abaxial side of its leaves but a marked reduction on the adaxial side, characterizing its leaves as terete. On the other hand, *T. pohliana* had the lowest density of scales on the abaxial face but the highest density on the adaxial face. Variation in scale distribution has been reported in other studies (Benzing, 2000) and, in both cases studied here, suggests a compensation mechanism that ensures efficient water absorption by the leaves. Although variation in scale density and distribution between the abaxial and adaxial leaf faces was recorded, all the collected plants were sympatric and grew under the same climatic conditions, which suggests that this is an intrinsic characteristic of each taxon. Previous studies have shown that scale density can be modulated by environmental factors, such as water availability and local rainfall, and other populations of *T. loliacea* exposed to lower rainfall levels showed an increase in scale density (Xavier and Arruda, 2025). However, the overall structure of the scales seems to be little influenced by environmental factors, such as precipitation (Cach-Pérez et al., 2016; Xavier and Arruda, 2025). Our results indicate that the observed structural traits are mainly intrinsic to each species, but minor environmental or anthropogenic influences in natural populations cannot be completely ruled out. It is important to note that our experimental population was derived from a single collection site. Consequently, while the observed traits appear to be largely species-specific, we cannot entirely exclude the possibility that population-specific genetic variation or unmeasured microenvironmental factors influenced the observed patterns. Although the total area of the scale and the area of the disc were similar between species, the total circularity was lower in *T. loliacea* than in *T. recurvata* and *T. pohliana*. This lower circularity, associated with the larger wing area in *T. loliacea*, may favor interception and the direction of water flow, thereby enhancing water uptake efficiency, as suggested by the higher C_max_ values observed for this species.

The differential distribution of pectins in peltate scales of *Tillandsia* indicates an intimate relationship with their function of water absorption. The presence of HGs with low and high levels of methyl esterification in the stalk (basal and dome cells) and in the epidermis in contact with the peltate scale suggests fine regulation of plasticity and cell adhesion. Low methyl-esterified HGs were strongly detected in the stalk cells, particularly in the regions contacting epidermal cells and on the outer faces of the disc where the wings are inserted. HGs are synthesized in a highly methyl-esterified form and can subsequently be demethyl-esterified by the action of PMEs (Albersheim et al., 2011). This dimethyl-esterification increases the availability of negatively charged HG residues capable of forming calcium-mediated cross-links. The resulting Ca²^+^ bridges promote the formation of the classical “egg-box” structure, thereby enhancing cell wall stiffening (Willats et al., 2001; Sénéchal et al., 2014). This configuration is consistent with the anchoring and attachment function of the peltate scale to the leaf ordinary epidermal cells, as regions with demethylated HGs tend to form more cohesive and less permeable networks (Mohen, 2008; Obomighie et al., 2025). In contrast, the presence of highly methyl-esterified HGs was more evident in the dome and wing cells, especially in *T. recurvata*, suggesting that these regions maintain greater cell wall flexibility. The high degree of methylation endows HGs with a lower calcium-binding capacity and greater hydration, characteristics associated with the elasticity and extensibility of the cell wall (Lionetti et al., 2010; Frey et al., 2024). Thus, in the wings, the predominance of highly methyl-esterified HGs may facilitate wing movement and maximize water uptake.

Epitopes of galactans and arabinans were detected in the peltate scales of *Tillandsia*, indicating an integrated arrangement between the rigidity and flexibility of the cell wall. Galactans, detected mainly in the cell walls of the stalks and in the junctions with the ordinary cells of epidermis, as well as in the wing insertion regions, indicate a role in the mechanical cohesion between the scale and the leaf surface. These polymers, which belong to the side chains of type I rhamnogalacturonans (RG-I), are associated with structural strength and cell anchorage, interacting with cellulose microfibrils and conferring stability during hydration and desiccation cycles (Varadarajan and Gilmartin, 1987; Zykwinska et al., 2005; Moneo-Sánchez et al., 2020), a typical condition of epiphytic species. On the other hand, the arabinans in the same areas seem to retain some cell wall flexibility, increasing the mobility of the pectic matrix, which may favor the transport of water and solutes (Ha et al., 2005; Zykwinska et al., 2005). The predominance of arabinans in association with highly methyl-esterified HGs in these regions suggests a dynamic and elastic interface between the scale and epidermis, whereas galactans and low-methylation HGs reinforce mechanical stability and structural adhesion.

Hemicelluloses detected in the cell junctions at the dome cells and at the insertion points of the scale with the ordinary cell of epidermis give strength and mechanical stability to the anchorage regions, especially in association with cellulose (Carpita and Gibeaut, 1993; Scheller and Ulvskov, 2010). The presence of an additional signal in the outer periclinal walls of the disc cells in *T. loliacea* allows structural rearrangements associated with variations in hydration (Cosgrove, 2005). The labeling of extensins and arabinogalactans (AGPs) in the peltate scales of the *Tillandsia* species suggests distinct but complementary roles in the architecture and dynamics of the cell wall. The extensins were detected in stalk cells, in the junctions with ordinary epidermal cells, and in the outer periclinal walls of the disc, indicating an active participation in the structural stabilization of the scale and in the anchoring with the ordinary epidermal cells. Extensins, which belong to the hydroxyproline-rich glycoprotein (HRGP) family, form cross-linked networks via covalent bonds between tyrosine residues, thereby contributing to mechanical reinforcement and resistance to abiotic stresses (Jamet et al., 2006). In addition, its deposition in regions of structural transition, such as the base of the scale, may be associated with the mechanical resistance during the hydration and desiccation cycles characteristic of *Tillandsia*. On the other hand, the AGPs distributed in stalk cells, discs, and junctions with epidermal cells appear to modulate wall plasticity. These proteins mediate interactions between the cell wall and the plasma membrane, participating in processes of cell adhesion, differentiation, and responses to environmental stimuli (Jamet et al., 2006). Considering the modulatory role of wall proteins in the reorganization of the noncellulosic and cellulosic matrix, it is possible that AGPs and extensins act in an integrated manner with expansins during scale development, promoting the loosening of cellulose microfibrils and allowing controlled flexibility and reconfiguration of the wall (Cosgrove, 2015).

The evident cuticle and the deposition of lipids in the anticlinal walls of the stalk cells can act as a hydrophobic film that directs the water flow between the wings and the ordinary cell of epidermis to the base of the scale toward the mesophyll, a pattern previously described in the literature (John and Hasenstein, 2017). The greater cuticle thickness observed in *T. recurvata* may reflect specific adaptations to minimize transpiration or regulate water entry and, in association with cell wall lignification, represent a dual strategy to direct water flow through the apoplast. The lignin in the basal cells reinforces anchorage in the leaf and provides a solid base for the movements observed at the scale (Ha et al., 2021).

### Leaf water absorption by peltate scales

The mechanism of water absorption by the peltate scales is based on the combination of structural and chemical properties that favor the uptake and capillary transport of water on the leaf surface. As indicated by the apoplastic marker LY, water adheres to the wings and rapidly spreads between the overlapping scales, then moves into the cells of the central disc and continues along the anticlinal axis until it is distributed throughout the internal tissues, as previously described in the literature (Stefano et al., 2008; Ha et al., 2021). In this study, *T. loliacea* had relatively large wings and high C_max_ values, indicating that big wings increase the uptake area. During this process, the microstructure alternates between hydrophilic and hydrophobic regions, directing water flow and reducing evaporation losses. Observations in *Pleopeltis polypodioides* indicate that upon hydration, the wings expand and curve toward the epidermal cells, creating a wick effect that moves water to living tissues (John and Hasenstein, 2017). Thus, the set of morphological and compositional attributes of the peltate scales constitutes a highly efficient system of foliar water uptake and conduction in environments with low water availability.

Another model proposed by Raux et al. (2020) suggests that the conspicuously thick-walled cells of the central disc function as the primary interface with the atmosphere for foliar water uptake, directing water to the remaining cells of the peltate scale rather than to the wings. In this case, we can highlight the chemical complexity of the outer periclinal thickening of disc cells, especially the pectins and hemicelluloses, which together can contribute to water flow. Our study showed a relationship between the circularity of the central disc and the maximum foliar water uptake capacity, in which, in individuals with less circularity (i.e., the disc was more misshapen), the C_max_ was higher. It is possible, then, that the two models can act together and need not be mutually exclusive.

The greater the water storage capacity (SWC) in tissues, the higher the rate of water absorption by the leaves, whereas an increase in the relative water content has a negative effect on the FWU. These findings corroborate several studies that have shown that under conditions of lower hydration, the water potential gradient between the air and the interior of the leaf favors the entry of water (Slatyer, 1960; Simonin et al., 2009; Oliveira R. S. et al., 2014; Vesala et al., 2017; Cernusak et al., 2018). Thus, leaf-atmospheric water flow occurs when the water potential gradient is inverted, with water potential higher in the atmosphere than inside the leaf, which explains why individuals with lower relative water contents had higher C_max_ values.

Although some epiphyte species have shown a trade-off between water absorption capacity and water storage capacity, that is, species with higher FWU have lower SWC, for example (Gotsch et al., 2015; Pan et al., 2021; Lima et al., 2023), the species analyzed in this study do not follow this pattern. Perhaps this is due to the low RWC values obtained (below 50%) compared with those of other epiphytes with succulent leaves (see, for example, Lima et al. (2023), in which all RWC values remain above 90%). In *T. loliacea*, *T. recurvata*, and *T. pohliana*, the water storage capacity was proportional to leaf absorption, indicating that the leaf succulence of these species does not affect the FWU. This pattern also differs from that of tank species such as *T. utriculata*, whose high storage capacity is associated with a reduced FWU but is sustained for a longer time (Berry et al., 2019; Rosado-Calderón et al., 2020). Thus, our results indicate that, for the studied *Tillandsia*, the FWU is not compromised by high storage capacity but rather an interesting alternative in the face of low momentary water content, optimizing the rapid replacement of water in short periods of atmospheric humidity.

From a morpho-functional perspective, the results indicate that the FWU can also be influenced by specific characteristics of peltate scales. Individuals with lower scale density and larger wing area may exhibit higher C_max_ values or influence water absorption rates, although in our study, significant functional relationships were observed only in *T. pohliana*. In this species, the abaxial density increased the water absorption rate, suggesting that greater scale cover favors the distribution and entry of water into the leaf (Brewer et al., 1991; Brewer and Smith, 1997). The scales showed similar total area and in the central disc area, but varied in circularity, generating overlapping points of the wings that modulate the flow and dispersion of water on the leaf. According to some authors, overlapping and more elaborate wings may favor the initial dispersion of water. This is observed in plants with a juvenile atmospheric phase that changes to a tank shape in the adult phase. For these individuals, the presence of elaborate and very intricate wings hinders the flow of water to the base, where it is absorbed, increasing water loss by evaporation (Benzing, 1970; 1976; 1980; Adams and Martin, 1986).

## Conclusion

The presence of pectins in the cell walls of the central disc, stalk, and leaf epidermis reinforces the role of these regions as water entry and conduction routes. Highly hydratable pectins form a hydrophilic matrix that facilitates capillary and osmotic transport between cells, allowing water to advance through the scales and epidermis. Hemicelluloses, especially xylans/arabinoxylans and heteromannans, directly contribute to the cohesion between cellulose microfibrils and mechanically reinforce the anchorage regions of the scale, allowing the structure to support the cycles of hydration and desiccation. Wall proteins, such as extensins and AGPs, modulate the rigidity and plasticity of the matrix, stabilizing contact zones with the epidermis and promoting fine structural adjustments during water uptake. In turn, the lignins reinforce resistance in the basal cells of the stalk, and together with the lipids, they play a complementary role by forming selective barriers that reinforce impermeability, direct apoplastic flow toward mesophyll cells, and reduce losses by evaporation. In addition, interspecific differences in scale density, distribution, and morphometry, as well as in cuticle thickness and parietal composition, demonstrate complementary strategies to optimize water absorption and retention.

## Data Availability

The raw data supporting the conclusions of this article will be made available by the authors, without undue reservation.
