# Supplementary material for: Functional architecture and cell wall composition of peltate scales involved in leaf water absorption
Source: Front Plant Sci. 2026 Feb 12;17:1756403. doi: 10.3389/fpls.2026.1756403 (PMC12935889; doi:10.3389/fpls.2026.1756403)
Supplement: Supplementary file 1 [file Table1.docx]

**Supplementary material**

Table 1 - Relationships among FWU parameters (maximum foliar water uptake capacity - C_max_, and the velocity of foliar water uptake - k) and leaf traits (relative water content, saturation water content, total area of the scale, scale circularity, disc area, disc circularity, wing area, scale density in the abaxial and adaxial face of the leaves) of the *Tillandsia loliacea*, *T. recurvata*, and *T. pohliana* leaves. The analysis considered all plants of the three different species.

| Dependent variable | Independent variable | R^2^ | Equation | F values | P value |
| --- | --- | --- | --- | --- | --- |
| C_max_ | Relative water content (RWC) | 0.65 | Y = -1.588*X + 89.42 | 24.28 | < 0.001 |
|  | Saturation water content (SWC) | 0.74 | Y = 5.823*X - 29.99 | 36.12 | < 0.0001 |
|  | Total area of the scale | 0.002 | Y = 83.65*X + 21.25 | 0.03 | > 0.05 |
|  | Scale circularity | 0.12 | Y = -39.48*X + 48.48 | 1.84 | > 0.05 |
|  | Disc area | 0.20 | Y = -5334*X + 117.9 | 3.34 | > 0.05 |
|  | Disc circularity | 0.48 | Y = -4449*X + 4456 | 11.88 | < 0.01 |
|  | Wing area | 0.02 | Y = 254.9*X + 9.612 | 0.21 | > 0.05 |
|  | Scale density in the abaxial face of the leaves | 0.09 | Y = -1.328*X + 72.21 | 1.41 | > 0.05 |
|  | Scale density in the adaxial face of the leaves | 0.37 | Y = -1.878*X + 93.34 | 4.79 | > 0.05 |
| k | Relative water content (RWC) | 0.04 | Y = 7.722e-005*X + 0.006849 | 0.51 | > 0.05 |
|  | Saturation water content (SWC) | 0.06 | Y = -0.0003473*X + 0.01331 | 0.89 | > 0.05 |
|  | Total area of the scale | 0.03 | Y = 0.06539*X + 0.003720 | 0.42 | > 0.05 |
|  | Scale circularity | 0.07 | Y = 0.005914*X + 0.006871 | 0.95 | > 0.05 |
|  | Disc area | 0.13 | Y = 0.8538*X - 0.004435 | 1.92 | > 0.05 |
|  | Disc circularity | 0.02 | Y = 0.1967*X - 0.1859 | 0.30 | > 0.05 |
|  | Wing area | 0.02 | Y = 0.05355*X + 0.005710 | 0.23 | > 0.05 |
|  | Scale density in the abaxial face of the leaves | 0.01 | Y = -6.962e-005*X + 0.01205 | 0.09 | > 0.05 |
|  | Scale density in the adaxial face of the leaves | 0.02 | Y = 7.476e-005*X + 0.006066 | 0.15 | > 0.05 |

Table 2- Relationships among FWU parameters (maximum foliar water uptake capacity - C_max_, and the velocity of foliar water uptake - k) and leaf traits (relative water content, saturation water content, total area of the scale, scale circularity, disc area, disc circularity, wing area, scale density in the abaxial and adaxial face of the leaves) of *Tillandsia loliacea* plants.

| Dependent variable | Independent variable | R^2^ | Equation | F values | P value |
| --- | --- | --- | --- | --- | --- |
| C_max_ | Relative water content (RWC) | 0.31 | Y = -1.578*X + 102.2 | 1.38 | > 0.05 |
|  | Saturation water content (SWC) | 0.69 | Y = -25.48*X + 335.7 | 6.95 | > 0.05 |
|  | Total area of the scale | 0.008 | Y = -108.5*X + 55.55 | 0.02 | > 0.05 |
|  | Scale circularity | 0.05 | Y = -40.51*X + 55.58 | 0.15 | > 0.05 |
|  | Disc area | 0.001 | Y = 153.9*X + 42.50 | 0.002 | > 0.05 |
|  | Disc circularity | 0.54 | Y = -2682*X + 2714 | 3.48 | > 0.05 |
|  | Wing area | 0.015 | Y = -182.8*X + 59.77 | 0.05 | > 0.05 |
|  | Scale density in the abaxial face of the leaves | 0.016 | Y = -0.3540*X + 56.87 | 0.05 | > 0.05 |
|  | Scale density in the adaxial face of the leaves | 0.28 | Y = -1.356*X + 89.35 | 1.22 | > 0.05 |
| k | Relative water content (RWC) | 0.90 | Y = 0.001179*X - 0.03598 | 26.65 | < 0.05 |
|  | Saturation water content (SWC) | 0.58 | Y = 0.01031*X - 0.1108 | 4.21 | > 0.05 |
|  | Total area of the scale | 0.24 | Y = 0.2591*X - 0.01837 | 0.94 | > 0.05 |
|  | Scale circularity | 0.50 | Y = -0.05882*X + 0.02212 | 3.01 | > 0.05 |
|  | Disc area | 0.03 | Y = 0.4317*X - 0.0002705 | 0.11 | > 0.05 |
|  | Disc circularity | 0.02 | Y = 0.2505*X - 0.2424 | 0.07 | > 0.05 |
|  | Wing area | 0.31 | Y = 0.3707*X - 0.02315 | 1.36 | > 0.05 |
|  | Scale density in the abaxial face of the leaves | 0.23 | Y = 0.0005896*X - 0.01295 | 0.89 | > 0.05 |
|  | Scale density in the adaxial face of the leaves | 0.05 | Y = -0.0002610*X + 0.01531 | 0.17 | > 0.05 |

Table 3- Relationships among FWU parameters (maximum foliar water uptake capacity - C_max_, and the velocity of foliar water uptake - k) and leaf traits (relative water content, saturation water content, total area of the scale, scale circularity, disc area, disc circularity, wing area, scale density in the abaxial and adaxial face of the leaves) of *Tillandsia pohliana* plants.

| Dependent variable | Independent variable | R^2^ | Equation | F values | P values |
| --- | --- | --- | --- | --- | --- |
| C_max_ | Relative water content (RWC) | 0.04 | Y = -0.03449*X + 5.060 | 0.14 | > 0.05 |
|  | Saturation water content (SWC) | 0.25 | Y = -1.208*X + 11.31 | 1.01 | > 0.05 |
|  | Total area of the scale | 0.05 | Y = 10.41*X + 2.344 | 0.16 | > 0.05 |
|  | Scale circularity | 0.03 | Y = -0.8472*X + 3.850 | 0.08 | > 0.05 |
|  | Disc area | 0.01 | Y = -3.698*X + 3.379 | 0.0002 | > 0.05 |
|  | Disc circularity | 0.03 | Y = 38.26*X - 34.66 | 0.11 | > 0.05 |
|  | Wing area | 0.06 | Y = 12.73*X + 2.331 | 0.20 | > 0.05 |
|  | Scale density in the abaxial face of the leaves | 0.15 | Y = 0.09352*X + 0.6507 | 0.53 | > 0.05 |
|  | Scale density in the adaxial face of the leaves | 0.004 | Y = 0.005440*X + 3.097 | 0.01 | > 0.05 |
| k | Relative water content (RWC) | 0.21 | Y = 0.0002173*X - 0.0001048 | 0.82 | > 0.05 |
|  | Saturation water content (SWC) | 0.0002 | Y = -8.865e-005*X + 0.01145 | 0.0005 | > 0.05 |
|  | Total area of the scale | 0.01 | Y = 0.01316*X + 0.009626 | 0.03 | > 0.05 |
|  | Scale circularity | 0.33 | Y = -0.008651*X + 0.01628 | 1.48 | > 0.05 |
|  | Disc area | 0.22 | Y = 0.5877*X + 0.001399 | 0.87 | > 0.05 |
|  | Disc circularity | 0.14 | Y = -0.2204*X + 0.2297 | 0.51 | > 0.05 |
|  | Wing area | 0.003 | Y = 0.007984*X + 0.01024 | 0.009 | > 0.05 |
|  | Scale density in the abaxial face of the leaves | 0.85 | Y = -0.0006369*X + 0.02903 | 18.16 | < 0.05 |
|  | Scale density in the adaxial face of the leaves | 0.15 | Y = -9.780e-005*X + 0.01487 | 0.54 | > 0.05 |

Table 4- Relationships among FWU parameters (maximum foliar water uptake capacity - C_max_, and the velocity of foliar water uptake - k) and leaf traits (relative water content, saturation water content, total area of the scale, scale circularity, disc area, disc circularity, wing area, scale density in the abaxial and adaxial face of the leaves) of *Tillandsia recurvata* plants.

| Dependent variable | Independent variable | R^2^ | Equation | F values | P values |
| --- | --- | --- | --- | --- | --- |
| C_max_ | Relative water content (RWC) | 0.54 | Y = -1.654*X + 84.01 | 3.59 | > 0.05 |
|  | Saturation water content (SWC) | 0.68 | Y = 3.877*X - 9.278 | 6.52 | > 0.05 |
|  | Total area of the scale | 0.68 | Y = -1297*X + 152.2 | 6.42 | > 0.05 |
|  | Scale circularity | 0.18 | Y = 82.83*X - 10.33 | 0.65 | > 0.05 |
|  | Disc area | 0.64 | Y = -7155*X + 164.2 | 5.34 | > 0.05 |
|  | Disc circularity | 0.07 | Y = 1913*X - 1870 | 0.24 | > 0.05 |
|  | Wing area | 0.62 | Y = -1413*X + 137.6 | 4.83 | > 0.05 |
|  | Scale density in the abaxial face of the leaves | 0.30 | Y = -1.512*X + 92.50 | 1.31 | > 0.05 |
|  | Scale density in the adaxial face of the leaves | -- | -- | -- | -- |
| k | Relative water content (RWC) | 0.15 | Y = 0.0002536*X + 0.004773 | 0.53 | > 0.05 |
|  | Saturation water content (SWC) | 0.37 | Y = -0.0008305*X + 0.02200 | 1.77 | > 0.05 |
|  | Total area of the scale | 0.23 | Y = 0.2196*X - 0.007497 | 0.90 | > 0.05 |
|  | Scale circularity | 0.39 | Y = -0.03563*X + 0.03282 | 1.91 | > 0.05 |
|  | Disc area | 0.55 | Y = 1.933*X - 0.02218 | 3.68 | > 0.05 |
|  | Disc circularity | 0.59 | Y = -1.589*X + 1.597 | 4.40 | > 0.05 |
|  | Wing area | 0.16 | Y = 0.2102*X - 0.002987 | 0.57 | > 0.05 |
|  | Scale density in the abaxial face of the leaves | 0.31 | Y = 0.0004454*X - 0.004123 | 1.35 | > 0.05 |
|  | Scale density in the adaxial face of the leaves | -- | -- | -- | -- |
